# The *Caenorhabditis elegans* THO Complex Is Required for the Mitotic Cell Cycle and Development

**DOI:** 10.1371/journal.pone.0052447

**Published:** 2012-12-20

**Authors:** Maikel Castellano-Pozo, Tatiana García-Muse, Andrés Aguilera

**Affiliations:** Department of Molecular Biology, Centro Andaluz de Biología Molecular y Medicina Regenerativa, Universidad de Sevilla, Sevilla, Spain; Texas A&M University, United States of America

## Abstract

THO is a conserved eukaryotic complex involved in mRNP biogenesis and RNA export that plays an important role in preventing transcription- and RNA-mediated genome instability in mitosis and meiosis. In mammals THO is essential for embryogenesis, which limits our capacity to analyze the physiological relevance of THO during development and in adult organisms. Using *Caenorhabditis elegans* as a model system we show that the THO complex is essential for mitotic genome integrity and the developmentally regulated mitotic cell cycles occurring during late postembryonic stages.

## Introduction

Maintenance of genome integrity is essential for development. Many genes controlling different cellular processes, including DNA replication, cell cycle progression, damage checkpoint, DNA repair and recombination, transcription and even RNA processing and export, have been implicated in the maintenance of genome stability in vegetatively growing cells [1]. Particularly intriguing are the roles that the THO and THSC/TREX-2 protein complexes involved in mRNP biogenesis and export have on genome integrity [2], [3].

THO is a multimeric complex composed of stoichiometric amounts of Tho2, Hpr1, Mft1, Thp2 and Tex1 as identified in *Saccharomyces cerevisiae* [4], [5]. It is conserved in all eukaryotes and the homologues have been identified in *Caenorhabditis elegans, Drosophila* and mammals (see [6]). Yeast cells lacking the THO complex show defects in transcription elongation and RNA export and a strong increase in transcription-associated recombination (TAR). Interestingly, TAR in these yeast mRNP biogenesis mutants and in vertebrate cells depleted of the ASF/SF2 splicing factor is linked to R-loops, similar to the process of class-switch recombination that occurs at the Immunoglobulin (Ig) genes in vertebrates [7]. In addition we have recently shown that *S. cerevisiae* and *C. elegans* THO mutants accumulate R-loops during meiosis causing pre-meiotic replication impairment as well as DNA damage accumulation [8].

In mammals, the THO complex THOC1 subunit is essential for embryogenesis, as determined by knocking out THOC1 subunit [9]. This has limited our capacity to analyze the physiological relevance of THO in development and in adult individuals. A model system to bypass these difficulties is *C. elegans*. Though THO is also essential in *C. elegans* maternal rescue permits the analysis of loss-of-function mutations in adult tissues of *thoc-2* null mutants [8]. Given the relevance of THO in RNA metabolism and genome integrity, we used *C. elegans* as a model system to analyze the role of *thoc-2* during development. Here we show that *thoc-2* mutants display general deficiencies during development, which we propose are due to defects in mitotic genome stability as a consequence of impaired replication. Our works reveals an essential role of an mRNP biogenesis factor in genome integrity maintenance during development.

## Materials and Methods

### Strains and Maintenance

Standard methods were used for the maintenance and manipulation of *C. elegans* strains [10]. The following nematode strains were provided by the *Caenorhabditis* Genetics Center, which is funded by the NIH National Center for Research Resources, including wild type Bristol N2, *thoc-2(ok961*), RB1164 [aly-2(ok1203)], RB805 [nfx-1&nfx-2 (ok611)] and JK2739. *thoc-2(tm1310)* was generated and kindly provided by S. Mitani.

Development analysis was done with synchronized L1 larvae that were followed through the four larvae stages up to 24 hours post-L4 adults. Images were taken with LEICA CA-MZ16 FA-FLUO.

Hydroxyurea (HU) treatment was performed as previously described [11]. L4 larvae were transferred to plates with 40 mM HU for 16 hours prior to analysis.

### Immunostaining and FISH

For all the antibodies used in this study but RNP-8 gonads were treated as described [12]. One day post-L4 adult gonads were dissected in PBS with levimasole on POLYSINE slides, fixed for 20 minutes in 4% paraformaldehyde and replaced for 10 minutes in TBSBTx (TBSB+0.4% TX100). The slides were washed twice for 10 minutes and one more for 30 minutes with TBSB (TBS+0.5% BSA). They were incubated overnight at 4°C with the antibody. Dilutions used: goat α-CEP-1 (1∶100), rabbit α-ATL-1 (1∶200), rat α-RPA-1 (1∶200), α-FK2 (1∶10000), rabbit α-GLD-1 (1∶100) [13], mouse α-SP-56 (1∶300), rabbit α-PGL-1 (1∶100) [14] (in TBSB). For rabbit α-RNP-8 (1∶1000) gonads were fixed (3% formaldehyde, 0.25% glutaraldehyde, 100 mM K2HPO4 [pH 7.2]). After three washes with PBT (PBS+0.1% Tween 20) gonads were treated with proteinase K (50 µg/ml) for 30 minutes at RT and then re-fixed in the same solution for 15 minutes at RT [15]. Next day gonads were washed 3 times in TBSB, each for 30 minutes at RT, and incubated for 2 hours with the secondary antibody in TBSB (αGOAT 1∶10000, αRABBIT 1∶5000, αRAT 1∶5000, αMOUSE 1∶5000). Gonads were washed three times for 30 minutes in TBSB and mounted with 10 µl Vectashield (with 1 µg/ml DAPI) per sample for further analysis.

FISH was performed as in [16] with modifications. Dissected gonads on slides were fixed in PGM (3% paraformaldehyde/0.25% glutaraldehyde/0.1 MK2HPO4 (pH 7.2)) for 1.5 to 2 hours at RT. A wash in PBTw (1XPBS+0.1% Tween20) was followed by the addition of 100% cold MeOH. Next, gonads were washed twice in PBTw. Then gonads were treated with 50 mg/ml Proteinase K in PBTw for 30 minutes at RT followed by three washes in PBTw. Fixation was repeated for 15 minutes in PGM followed by 15 minutes incubation in PBTw containing 2 mg/ml glycine. After three washes in PBTw, 50% PBTw/50% hybridization buffer was added (HB: 5X SSC, 50% deionized formamide, 100 mg/ml autoclaved Herring sperm DNA, 50 mg/ml Heparin and 0.1% Tween-20) and incubated 5 minutes at 50°C. Next a pre-hybridization step was performed adding HB and incubating for 1 hour at 50°C. For hybridization the probe against the poly-A sequence of mRNA (40×T) labeled with the Cy3 fluorochrome (Sigma) was added to pre-warmed HB at 10 µM, and gonads were incubated for 20 hour at 50°C. The following day samples were washed 4 times 15 minutes with pre-warmed HB at 50°C. Then the following washes were performed:10 minutes in 50% HB/50% PBTw, 2 times for 1 minute in PBTw, 3 times for 10 minutes in PBTw and 5 minutes in PBTw, all washes were performed at RT. Finally slides were mounted with 10 µl Vectashield (with 1 µg/ml DAPI) per sample for further analysis. Slides were kept at all times in a humid chamber.

### *In situ* Detection of Germline DNA Synthesis

Direct incorporation of Cy3-dUTP (Amersham Bioscences) into germline nuclei was performed as described [8]. The injection mix consisted of 50 µM Cy3-dUTP (Amersham Bioscences, Piscataway, NJ) in PBS, pH 7.2. After ∼2.5 hours of exposure to the Cy3-dUTP, gonads were dissected, fixed and DAPI-stained. The total number of cells that incorporated Cy3-dUTP was determined for each dissected germline. For both strains, N2 and *thoc-2*(*tm1310*), n = 28. Caffeine was co-microinjected at 0.05 mM. For both strains, N2 and *thoc-2*(*tm1310*), n = 15.

### Fluorescence Microscopy

Leica DM6000B inverted microscope was used to examine the germlines with 40X HCXPL-APO/1.25 OIL or 63X HCXPL-APO/1.40 OIL lens, and images captured using Leica LAS-AF computer software. Three-dimensional data sets were computationally deconvolved, and regions of interest then projected into one dimension.

### Quantitative RT-PCR

For each sample, about 100 adult worms were collected and total RNA was extracted using Rneasy MiniKit (Qiagen). 360 ng of total RNA from each sample were used for reverse transcription (Quantitect Reverse Transcription, Qiagen) and PCR using the Power SYBR® Green PCR-MasterMix (Invitrogen). The *cdc-42* gene, whose expression levels are unusually stable during development, was used as an endogenous standard. The oligos used were: 5′-CTTTGAGCAATGATGCGAAA-3′ and 5′-TCATTCGAGAATGTCCGAGA-3′ for *cdc-42*; 5′-GTGAGTGTGACGACGGATTT-3′ and 5′-CAGAAGAATAGCCAGCGAGA-3′ for *spe-9*; 5′-CGCATTTACAACGAGTGGAA-3′ and 5′-TGGATTGAGATGAGGAGCAG-3′ for *spe-15*; 5′-CCACCGCTTAATTTGATTCTT-3′ and 5′-TGCCTGATCCTCAACATCAT-3′ for *spe-41*. Experiments and data analysis were performed with a 7500-RealTime PCR System (Applied Biosystems).

## Results and Discussion

### *C. elegans thoc-2* Mutants Fail to Reach Adult Size

**Figure 1:**
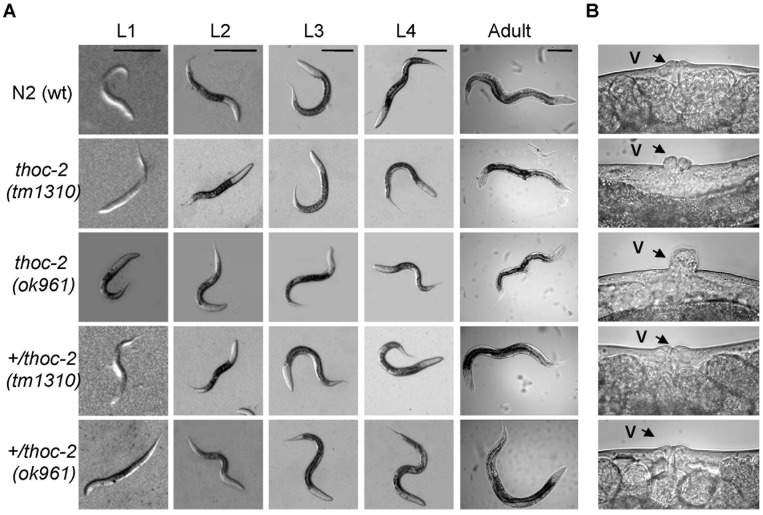
Development defects in *thoc-2* mutants. (**A**) Representative images of animals of the indicated genotype from the four larva stages and the adult stage at 24 hours post-L4. Scale bars represent 0.1 mm. (**B**) Body detail of animals of the indicated genotype. The arrow indicates the vulva.

The THO complex is conserved in yeast and metazoans [6], in Table S1 we summarize the putative components of the *C. elegans* THO complex based on sequence comparison [17]. One member of *C. elegans* THO complex is *thoc-2* (THO Complex subunit 2) ortholog of yeast *THO2*. We previously showed that *thoc-2* is essential for fertility but homozygous mutants produced by self-fertilization of heterozygous hermaphrodite parents can be analyzed [8]. We have characterized two *thoc-2* deletion mutant alleles, *thoc-2*(*tm1310*) and *thoc-2*(*ok961*), which are predicted to encode non-functional truncated proteins. Phenotypic analysis of *thoc-2* mutants reveals different developmental abnormalities. As shown in Figure 1A, homozygous worms of two different *thoc-2* deletion alleles develop from L1 to L4 normally, but show defects in reaching adulthood size. Consistent with a defect in completing development, a protruding vulva (Pvl) phenotype was observed in both *thoc-2* mutants with 100% penetrance, meaning that all mutant worms observed showed Pvl (Figure 1B). Development of the vulva requires several organized mitotic divisions of the three precursors cells [18], and it has been shown that loss of vulva cells by non-apoptotic cell death results in abnormal vulva phenotypes including Pvl [19]. Notably, defects in the vulva cell lineage of *C. elegans* have been related to mutations causing chromosome instability [20], [21]. Based on this, we hypothesized that *C. elegans thoc-2* mutants might also show defects in chromosome stability during mitotic divisions.

### Abnormal Mitosis in *thoc-2* Mutants

**Figure 2:**
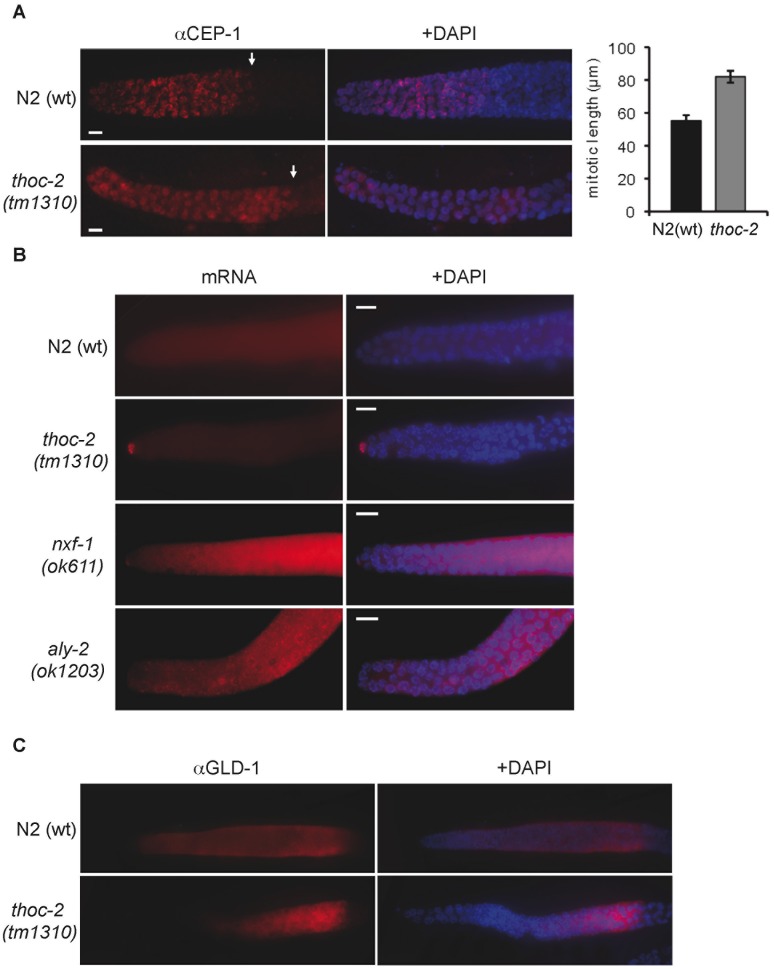
Defective mitosis in *thoc-2* mutants. (**A**) Representative images of fixed mitotic nuclei from N2(wt) and *thoc-2(tm1310)* animals immunostained with the mitotic marker CEP-1, and quantification of the mitotic region length. Error bars indicate standard error of mean (n = 15). (**B**) Poly-A mRNA visualized by FISH using a poly-dT oligonucleotide conjugated with Cy3 in germlines from animals of the indicated genotype. (**C**) Representative images of fixed mitotic nuclei from animals of the indicated genotype immunostained with GLD-1.

To determine whether mitosis was affected, we analyzed *thoc-2* mutant germlines. Each germline is spatially polarized in a distal to proximal manner with respect to mitotic proliferation and progression through meiotic prophase I. In the distal region nuclei proliferate by mitotic divisions acting as stem cells until they reach the transition zone. Here, following premeiotic S-phase, initial pairing events between homologous chromosomes take place and can be recognized by the polarized redistribution of chromosomes that give rise to “crescent” shaped nuclei [22]. We measured the length of the mitotic region in N2(wt) and *thoc-2* mutant strains by the shape of the transition nuclei and by immunofluorescence with the CEP-1 mitosis marker [23]. The two *thoc-2* deletion mutants analyzed showed longer mitotic regions expanded by approximately 30 µm as compared to the mitotic region length in N2(wt) animals (Figure 2A and Figure S1A).

In adults, the distal tip cell (DTC) located at the tip of the mitotic region controls the mitosis-meiosis transition. The DTC forms a stem cell niche via the Notch signaling pathway, which in turn controls a complex network of several proteins and mRNAs, including GLD-1, GLD-2, FBF-1/2 and NOS-3, all of which are involved in post-transcriptional gene regulation that finally regulates the mitosis-meiosis transition. The DTC has high rates of mRNA synthesis. Since THO mutants show mRNA export defects, we examined the mRNA accumulation in *thoc-2* mutants. FISH analysis with an oligo-dT probe against poly-A showed a striking accumulation of mRNA at the DTC of *thoc-2* mutant germlines in contrast to N2(wt) germlines where no signal was distinguishable (Figure 2B and Figure S1B). To address if this was a general phenotype of mRNA biogenesis mutants we also tested *nfx-1* and *aly-2* mutants [24], [25]. NFX-1 and ALY-2 are homologues of the evolutionary conserved *S. cerevisiae* mRNA export factors Mex67 and Yra1, respectively [26]. In agreement with their role, mutants of both genes show mRNA accumulation along germline nuclei (Figure 2B and Figure S1C). However, no mRNA accumulation was detected in the DTC of *aly-2* mutants germlines, and only a slight signal was observed in the *nfx-1* DTC (Figure 2B). This is in agreement with the fact that no extended mitotic regions are present in neither of both mutants (Figure S1A). To further examine the relationship between mRNA accumulation and mitotic defects in *thoc-2* mutants, we decided to analyze the distribution of GLD-1 [13]. GLD-1 is a key member of the mitosis-meiosis transition regulatory network (reviewed in [27]). Using anti-GLD-1 antibodies we observed that GLD-1 accumulation in the *thoc-2* mutants was delayed as compared to N2(wt), but increased gradually in the proximal part of the mitotic region and reached the highest level as germ cells entered the meiotic prophase (Figure 2C). This shifted distribution can explain the long mitotic region of *thoc-2* mutants, as it takes longer for GLD-1 to reach the level necessary to trigger meiosis. It has been shown that meiotic entry prevention can be regulated, at least in part, by lowering GLD-1 abundance [28].

Importantly, mRNA accumulation at the DTC does not affect the initial steps of meiosis, as the synaptonemal complex is normal in *thoc-2* mutants [8]. In addition, analysis of the expression and localization of meiotic markers involved in oogenesis or spermatogenesis showed no differences between N2(wt) and *thoc-2* mutants (see [15], [29]–[31] and Figure S2). Taken together these data suggest that there are tissue specific gene products required for mRNA biogenesis that would explain the different development patterns caused by loss of the THO complex.

### Checkpoint Activation in *thoc-2* Mutants

**Figure 3:**
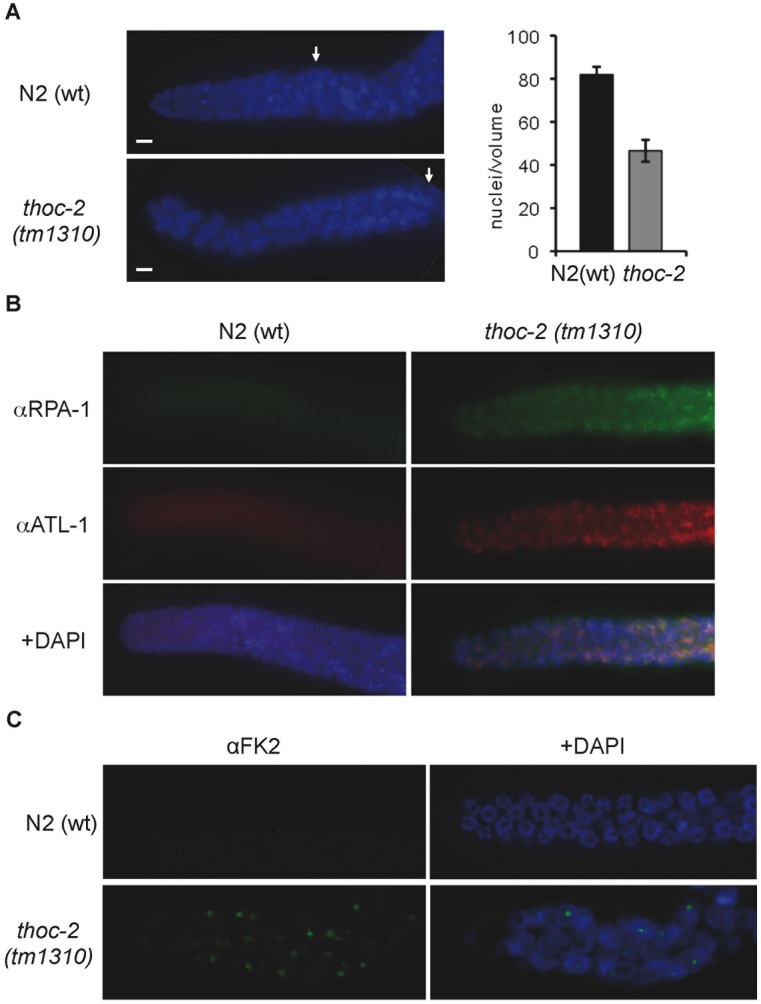
Mitotic DNA damage activation and replication impairment in *thoc-2* mutants. (**A**) Representative images of a single focal plane through the mitotic region of the germline from N2(wt) and *thoc-2(tm1310)* counterstained with DAPI, and quantification of the number of mitotic nuclei. Error bars indicate standard errors of means (n = 20). (**B**) Representative images of fixed mitotic nuclei from animals of the indicated genotype immunostained with antibodies against the replication protein RPA and the checkpoint kinase ATL-1. (**C**) Representative images of fixed mitotic nuclei from animals of the indicated genotype immunostained with FK-2.

Notably, despite the long mitotic regions, the total number of nuclei in the *thoc-2* mutants was reduced as compared to wild type (Figure 3A and Figure S1A). Cytological analysis of the *thoc-2* germlines also revealed the presence of enlarged nuclei, a hallmark of cell cycle arrest. To further analyze the disorganized mitotic nuclei we used antibodies against the single-strand binding protein RPA, which is involved in replication and recombination. Unlike wild type worms, *thoc-2* mutants exhibited regions of ssDNA as indicated by the presence of RPA foci (Figure 3B). This phenotype is partially similar to that observed in mutants with defects during S-phase such as *atl-1* and *clk-2*, which show RPA foci in mitotic nuclei [11], [32]. This suggests that the accumulated ssDNA likely triggered the observed cell cycle arrest. In order to determine if the DNA damage checkpoint was triggered in the *thoc*-2 mutants we used antibodies against the checkpoint protein ATL-1. ATL-1 is recruited to sites of DNA damage where it activates the DNA damage checkpoint and it is required for mitotic cell cycle arrest [32]. No signal was observed in the N2(wt) animals but *thoc-2* mutants showed ATL-1 foci in mitotic nuclei (Figure 3B), consistent with the interpretation that ssDNA is activating the DNA damage checkpoint in *thoc-2* mutants. This implies that the absence of THOC-2 hinders the S-phase, leading to an accumulation of ssDNA that activates the S-phase checkpoint. Treatment of N2 (wt) with the inhibitor of ribonucleotide reductase that leads to replication fork stalling, HU, induces S-phase arrest that manifests as a reduction in the number of nuclei in the mitotic compartment of the germline as well as an increase in mitotic region length [11], similar to what we observe in *thoc-2* mutants mitotic regions (Figure 2A and 3A). We determined the replication stress response in *thoc-2* mutants by treatment with hydroxyurea (HU). HU treatment of N2 (wt) worms leads to the normal response (Figure S3), in *thoc-2* mutants we observed a slight reduction in the number of mitotic nuclei (Figure S3), confirming that the *thoc-2* mitotic nuclei are under replicative stress. After checkpoint activation many proteins are ubiquitynilated during DNA damage repair [33]. As a way to test if DNA repair processes are taking place in *thoc-2* mutants we used FK2 antibody against conjugated Ubiquitin (Ub). Contrary to N2(wt) animals, *thoc-2* mutants showed FK2 foci in mitotic nuclei (Figure 3C), indicating that DNA damage in *thoc-2* mutants is activating the repair pathways. All together these observations indicate a role for *thoc-2* in the maintenance of normal mitosis in the germline.

The relevance of S-phase checkpoint activation in vegetatively growing THO mutants was reported in *S. cerevisiae* [34]. Double mutants of *hpr1* with mutations in the structural gene of Rad24 and other components of the RFC-like complex involved in S-phase checkpoint activation either show a synthetic growth defect or are synthetic lethal under replication stress. In *hpr1*Δ the S-phase checkpoint activation is due to R-loops accumulation as shown by the separation-of-function *hpr1-101* mutant, which does not accumulate R-loops [35]. Contrary to *hpr1*Δ mutants, *hpr1-101* mutant does not activate the S-phase checkpoint since no Rad53 phosphorylation can be detected [35]. Consistent with these results, we observed checkpoint activation in the germline mitotic cells of *C. elegans thoc-2* mutants that also accumulate R-loops [8], which suggests that the importance of THO complex in S-phase progression can be extended from yeast to other eukaryotes.

### *thoc-2* and DNA Replication

**Figure 4:**
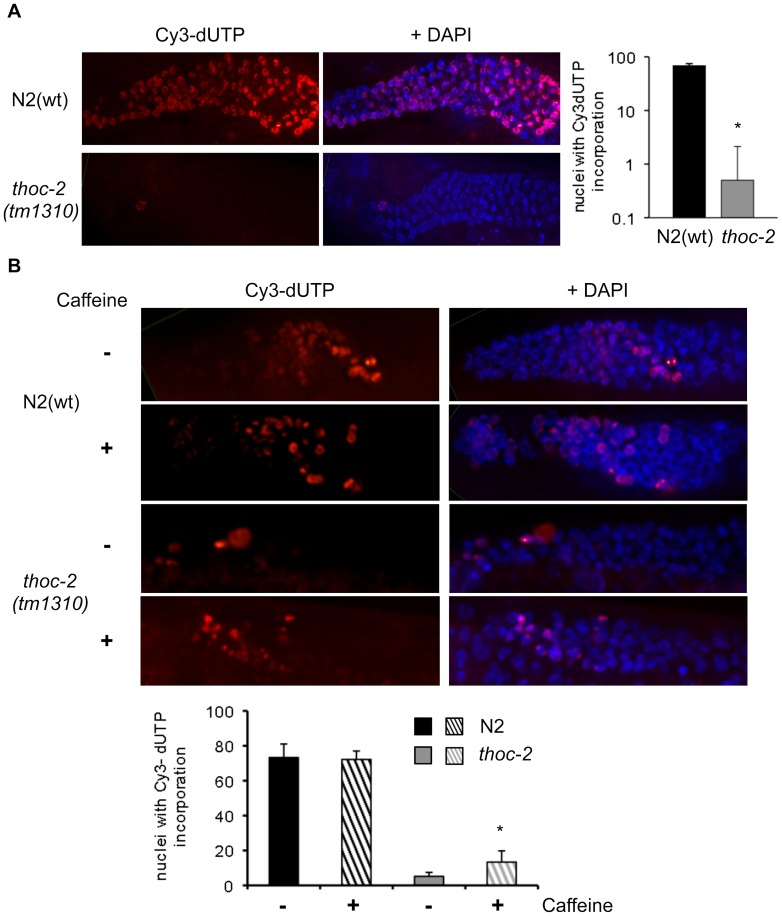
Mitotic replication is impaired in *C. elegans thoc-2* germlines and partially alleviated by checkpoint inhibition. (**A**) Representative images of fixed germlines of the indicated genotype of adult hermaphrodites 2.5 hours after microinjection with Cy3-dUTP and quantification of Cy3-dUTP incorporation. Error bars indicate standard error of mean (n = 28). Statistically significant differences versus the N2(wt) (p<0.001) is indicated by an asterisk * (Student’s t-Test). (**B**) Representative images of fixed germlines of the indicated genotype of adult hermaphrodites after microinjection with Cy3-dUTP with or without caffeine and quantification of Cy3-dUTP incorporation. Statistically significant differences versus untreated (p<0.001, n = 15) is indicated by an asterisk * (Student’s t-Test).

In *C. elegans* DNA replication failure and DNA lesions lead to prolonged cell cycle arrest of mitotic germ cells and the occurrence of RPA and ATL-1 foci throughout the germline [32], [36]. Therefore, we wondered whether the origin of mitotic genome instability in *C. elegans thoc-2* mutants would occur during the replication process. To examine replication, we assessed incorporation of deoxyribonucleotides into germline DNA as previously described [37], [38]. Following microinjection of Cy3-dUTP into young adult hermaphrodite germlines, worms were allowed to recover for 2.5 hours, after which their germlines were dissected, fixed and DAPI-stained. Quantification of the number of nuclei that incorporated Cy3-dUTP in each germline revealed that N2(wt) animals showed Cy3-dUTP incorporation in mitotic nuclei (Figure 4A), similar to previous reports [38]. In contrast, there was a significant reduction in the level of co-localization of Cy3-dUTP with DAPI staining in *thoc-2* mutant germlines (Figure 4A). This result clearly demonstrates that mitotic replication is impaired in *thoc-2* mutants. To test if cell cycle arrest was responsible of the reduced levels of replication we co-microinjected caffeine, an inhibitor of ATL-1. Co-microinjection of caffeine did not affect the incorporation of Cy3-dUTP in N2(wt) worms, whereas in *thoc-2* mutants it caused a slight increase in Cy3-dUTP-labelled nuclei (Figure 4B). Together the results suggest that the absence of THOC-2 hinders the S-phase. Endogenous DNA breaks can arise from replication defects, leading to the accumulation of ssDNA as indicated by the presence of RPA foci. As a consequence the S-phase checkpoint is activated, as confirmed by the ATL-1 foci observed in mitotic nuclei, and the cell cycle is arrested, which reduces the number of nuclei undergoing mitosis. The checkpoint dependence can be explained by the fact that THO mutants accumulate R-loops that, in turn, alter replication and become a source of DNA damage [8], [39], [40].

### Conclusions

We have exploited *Caenorhabditis elegans* as a model system to uncover the role of the THO complex in a multicellular organism. We have show by analysis of two deletions of the *thoc-2* component of THO, that the THO complex is required for completion of development. Our results reveal that the role of THO in mitotic genome stability maintenance is critical as shown by the protruding vulva and germline mitotic defects. We have shown that the DNA replication impairment of *thoc-2* mutants leads to DNA damage accumulation and checkpoint activation, with consequences in the progression through mitosis. Therefore, the lethality observed in *C. elegans* and mice are thus likely related to the incapacity of early embryonic cells to divide properly [8], [9]. In this sense the relevance of DNA replication and repair functions that warrants the integrity of the genome and proper progression through S-phase has been reported with the embryonic lethality of knock-out mice for DSB repair genes such as *RAD51* [41]. Our results also show the specific requirement of THO complex for mRNA export at the DTC. It is possible that THO mutants impact in mRNA-transcription and -export is responsible for the failure of the mutants to reach adult size. However, a more likely explanation is that this defect arises from the impairment of mitotic division during development. Our work in *C. elegans* provides new insights to the THO complex contribution to the maintenance of genome integrity in developmental mitotic divisions.

## Supporting Information

Figure S1 **Normal mitosis in** ***thoc-2*** **heterozygous worms.** (**A**) Representative images of a single focal plane through the mitotic region of the germline from animals of the indicated genotype counterstained with DAPI. (**B**) Accumulation of mRNA visualized by FISH with a poly-dT oligonucleotide conjugated with Cy3 in germlines from animals of the indicated genotype image at the mitotic region. (**C**) Accumulation of mRNA visualized by FISH with a poly-dT oligonucleotide conjugated with Cy3 in germlines from animals of the indicated genotype image at the pachytene region.
(PDF)

Figure S2 **Normal expression and localization of meiosis markers in** ***thoc-2*** **mutants.** (**A**) Representative images of fixed pachytene nuclei from N2(wt) and *thoc-2(tm1310)* worms immunostained with the oogenesis marker PGL-1. (**B**) Representative images of fixed pachytene nuclei from N2(wt) and *thoc-2(tm1310)* worms immunostained with the oogenesis marker RNP-8. (**C**) Representative images of fixed pachytene nuclei from N2(wt) and *thoc-2(tm1310*) immunostained with the SP56 spermatogenesis marker. (**D**) RT-qPCR quantification of the indicated genes involved in subsequent steps of spermatogenesis in N2(wt) and thoc-2 mutant worms.
(PDF)

Figure S3 **Response to replication stress in** ***thoc-2*** **.** Representative images of a single focal plane through the mitotic region of the germline from animals of the indicated genotype counterstained with DAPI and quantification of the number of mitotic nuclei and the mitotic region length. Error bars indicate standard errors of means (n = 30). Statistically significant differences versus the untreated N2(wt) (p<0.001) is indicated by an asterisk * (Student’s t-Test).
(PDF)

Table S1 *C. elegans* THO complex comparison with mammalian THO complex members.
(PDF)

## References

[1] Aguilera A, Gómez-González B (2008) Genome instability: a mechanistic view of its causes and consequences. Nat Rev Genet 9: 204–217 doi:10.1038/nrg2268. 18227811 10.1038/nrg2268

[2] Jimeno S, Rondon AG, Luna R, Aguilera A (2002) The yeast THO complex and mRNA export factors link RNA metabolism with transcription and genome instability. EMBO J 21: 3526–3535 doi:10.1093/emboj/cdf335. 12093753 10.1093/emboj/cdf335 PMC126085

[3] Luna R, Jimeno S, Marin M, Huertas P, Garcia-Rubio M, et al (2005) Interdependence between transcription and mRNP processing and export, and its impact on genetic stability. Mol Cell 18: 711–722 doi:10.1016/j.molcel.2005.05.001. 15949445 10.1016/j.molcel.2005.05.001

[4] Chavez S, Beilharz T, Rondon AG, Erdjument-Bromage H, Tempst P, et al (2000) A protein complex containing Tho2, Hpr1, Mft1 and a novel protein, Thp2, connects transcription elongation with mitotic recombination in *Saccharomyces cerevisiae* . EMBO J 19: 5824–5834 doi:10.1093/emboj/19.21.5824. 11060033 10.1093/emboj/19.21.5824 PMC305808

[5] Peña A, Gewartowski K, Mroczek S, Cuéllar J, Szykowska A, et al (2012) Architecture and nucleic acids recognition mechanism of the THO complex, an mRNP assembly factor. EMBO J 31: 1605–1616 doi:10.1038/emboj.2012.10. 22314234 10.1038/emboj.2012.10 PMC3321177

[6] Luna R, Rondon AG, Aguilera A (2011) New clues to understand the role of THO and other functionally related factors in mRNP biogenesis. Biochim Biophys Acta. doi:10.1016/j.bbagrm.2011.11.012. 10.1016/j.bbagrm.2011.11.012 22207203

[7] Aguilera A, Garcia-Muse T (2012) R loops: from transcription byproducts to threats to genome stability. Mol Cell 46: 115–124 doi:10.1016/j.molcel.2012.04.009. 22541554 10.1016/j.molcel.2012.04.009

[8] Castellano-Pozo M, Garcia-Muse T, Aguilera A (2012) R-loops cause replication impairment and genome instability during meiosis. EMBO Rep. doi:10.1038/embor.2012.119. 10.1038/embor.2012.119 PMC3463965 22878416

[9] Wang X, Chang Y, Li Y, Zhang X, Goodrich DW (2006) Thoc1/Hpr1/p84 is essential for early embryonic development in the mouse. Mol Cell Biol 26: 4362–4367 doi:10.1128/MCB.02163-05. 16705185 10.1128/MCB.02163-05 PMC1489088

[10] Brenner S (1974) The genetics of *Caenorhabditis elegans* . Genetics 77: 71–94. 4366476 10.1093/genetics/77.1.71 PMC1213120

[11] Ahmed S, Alpi A, Hengartner MO, Gartner A (2001) *C. elegans* RAD-5/CLK-2 defines a new DNA damage checkpoint protein. Curr Biol 11: 1934–1944. 11747819 10.1016/s0960-9822(01)00604-2

[12] Martin JS, Winkelmann N, Petalcorin MIR, McIlwraith MJ, Boulton SJ (2005) RAD-51-dependent and -independent roles of a *Caenorhabditis elegans* BRCA2-related protein during DNA double-strand break repair. Mol Cell Biol 25: 3127–3139 doi:10.1128/MCB.25.8.3127-3139.2005. 15798199 10.1128/MCB.25.8.3127-3139.2005 PMC1069622

[13] Suh N, Crittenden SL, Goldstrohm A, Hook B, Thompson B, et al (2009) FBF and its dual control of gld-1 expression in the *Caenorhabditis elegans* germline. Genetics 181: 1249–1260 doi:10.1534/genetics.108.099440. 19221201 10.1534/genetics.108.099440 PMC2666496

[14] Kawasaki I, Amiri A, Fan Y, Meyer N, Dunkelbarger S, et al (2004) The PGL family proteins associate with germ granules and function redundantly in *Caenorhabditis elegans* germline development. Genetics 167: 645–661 doi:10.1534/genetics.103.023093. 15238518 10.1534/genetics.103.023093 PMC1470885

[15] Kim KW, Nykamp K, Suh N, Bachorik JL, Wang L, et al (2009) Antagonism between GLD-2 binding partners controls gamete sex. Developmental Cell 16: 723–733 doi:10.1016/j.devcel.2009.04.002. 19460348 10.1016/j.devcel.2009.04.002 PMC2728548

[16] Lee MH, Schedl T (2006) RNA in situ hybridization of dissected gonads. WormBook: 1–7. doi:10.1895/wormbook.1.107.1. 10.1895/wormbook.1.107.1 PMC4781440 18050448

[17] Shaye DD, Greenwald I (2011) OrthoList: a compendium of *C. elegans* genes with human orthologs. PLoS ONE 6: e20085 doi:10.1371/journal.pone.0020085. 21647448 10.1371/journal.pone.0020085 PMC3102077

[18] Sternberg PW (2005) Vulval development. WormBook. doi:10.1895/wormbook.1.6.1. 10.1895/wormbook.1.6.1 PMC4781130 18050418

[19] Weidhaas JB, Eisenmann DM, Holub JM, Nallur SV (2006) A *Caenorhabditis elegans* tissue model of radiation-induced reproductive cell death. Proc Natl Acad Sci USA 103: 9946–9951 doi:10.1073/pnas.0603791103. 16788064 10.1073/pnas.0603791103 PMC1502559

[20] O’Connell KF, Leys CM, White JG (1998) A genetic screen for temperature-sensitive cell-division mutants of *Caenorhabditis elegans* . Genetics 149: 1303–1321. 9649522 10.1093/genetics/149.3.1303 PMC1460235

[21] McLellan J, O’Neil N, Tarailo S, Stoepel J, Bryan J, et al (2009) Synthetic lethal genetic interactions that decrease somatic cell proliferation in *Caenorhabditis elegans* identify the alternative RFC CTF18 as a candidate cancer drug target. Mol Biol Cell 20: 5306–5313 doi:10.1091/mbc.E09-08-0699. 19846659 10.1091/mbc.E09-08-0699 PMC2793303

[22] Garcia-Muse T, Boulton SJ (2007) Meiotic recombination in *Caenorhabditis elegans* . Chromosome Res 15: 607–621 doi:10.1007/s10577-007-1146-x. 17674149 10.1007/s10577-007-1146-x

[23] Schumacher B, Hanazawa M, Lee MH, Nayak S, Volkmann K, et al (2005) Translational repression of *C. elegans* p53 by GLD-1 regulates DNA damage-induced apoptosis. Cell 120: 357–368 doi:10.1016/j.cell.2004.12.009. 15707894 10.1016/j.cell.2004.12.009

[24] Kuersten S, Segal SP, Verheyden J, LaMartina SM, Goodwin EB (2004) NXF-2, REF-1, and REF-2 affect the choice of nuclear export pathway for *tra-2* mRNA in *C. elegans* . Mol Cell 14: 599–610 doi:10.1016/j.molcel.2004.05.004. 15175155 10.1016/j.molcel.2004.05.004

[25] Okkema P (2005) Transcriptional regulation. WormBook. doi:10.1895/wormbook.1.45.1. 10.1895/wormbook.1.45.1 PMC4781111 18050428

[26] Köhler A, Hurt E (2007) Exporting RNA from the nucleus to the cytoplasm. Nat Rev Mol Cell Biol 8: 761–773 doi:10.1038/nrm2255. 17786152 10.1038/nrm2255

[27] Kimble J, Crittenden SL (2005) Germline proliferation and its control. WormBook: 1–14. doi:10.1895/wormbook.1.13.1. 10.1895/wormbook.1.13.1 PMC4781503 18050413

[28] Jeong J, Verheyden JM, Kimble J (2011) Cyclin E and Cdk2 control GLD-1, the mitosis/meiosis decision, and germline stem cells in *Caenorhabditis elegans* . PLoS Genet 7: e1001348 doi:10.1371/journal.pgen.1001348. 21455289 10.1371/journal.pgen.1001348 PMC3063749

[29] Ward S, Roberts TM, Strome S, Pavalko FM, Hogan E (1986) Monoclonal antibodies that recognize a polypeptide antigenic determinant shared by multiple *Caenorhabditis elegans* sperm-specific proteins. The Journal of Cell Biology 102: 1778–1786. 2422180 10.1083/jcb.102.5.1778 PMC2114204

[30] L’Hernault SW (2006) Spermatogenesis. WormBook: 1–14. doi:10.1895/wormbook.1.85.1. 10.1895/wormbook.1.85.1 PMC4781361 18050478

[31] Kawasaki I, Shim YH, Kirchner J, Kaminker J, Wood WB, et al (1998) PGL-1, a predicted RNA-binding component of germ granules, is essential for fertility in *C. elegans* . Cell 94: 635–645. 9741628 10.1016/s0092-8674(00)81605-0

[32] Garcia-Muse T, Boulton SJ (2005) Distinct modes of ATR activation after replication stress and DNA double-strand breaks in *Caenorhabditis elegans* . EMBO J 24: 4345–4355 doi:10.1038/sj.emboj.7600896. 16319925 10.1038/sj.emboj.7600896 PMC1356337

[33] Polanowska J, Martin JS, Garcia-Muse T, Petalcorin MIR, Boulton SJ (2006) A conserved pathway to activate BRCA1-dependent ubiquitylation at DNA damage sites. EMBO J 25: 2178–2188 doi:10.1038/sj.emboj.7601102. 16628214 10.1038/sj.emboj.7601102 PMC1462971

[34] Gomez-Gonzalez B, Felipe-Abrio I, Aguilera A (2009) The S-phase checkpoint is required to respond to R-loops accumulated in THO mutants. Mol Cell Biol 29: 5203–5213 doi:10.1128/MCB.00402-09. 19651896 10.1128/MCB.00402-09 PMC2747986

[35] Gomez-Gonzalez B, Aguilera A (2009) R-loops do not accumulate in transcription-defective *hpr1–101* mutants: implications for the functional role of THO/TREX. Nucleic Acids Res 37: 4315–4321 doi:10.1093/nar/gkp385. 19451165 10.1093/nar/gkp385 PMC2715242

[36] Gartner A, Milstein S, Ahmed S, Hodgkin J, Hengartner MO (2000) A conserved checkpoint pathway mediates DNA damage–induced apoptosis and cell cycle arrest in *C. elegans* . Mol Cell 5: 435–443. 10882129 10.1016/s1097-2765(00)80438-4

[37] Jaramillo-Lambert A, Ellefson M, Villeneuve AM, Engebrecht J (2007) Differential timing of S phases, X chromosome replication, and meiotic prophase in the *C. elegans* germ line. Developmental Biology 308: 206–221 doi:10.1016/j.ydbio.2007.05.019. 17599823 10.1016/j.ydbio.2007.05.019

[38] Grigsby IF, Rutledge EM, Morton CA, Finger FP (2009) Functional redundancy of two *C. elegans* homologs of the histone chaperone Asf1 in germline DNA replication. Developmental Biology 329: 64–79 doi:10.1016/j.ydbio.2009.02.015. 19233156 10.1016/j.ydbio.2009.02.015

[39] Huertas P, Aguilera A (2003) Cotranscriptionally formed DNA:RNA hybrids mediate transcription elongation impairment and transcription-associated recombination. Mol Cell 12: 711–721. 14527416 10.1016/j.molcel.2003.08.010

[40] Domínguez-Sánchez MS, Barroso S, Gómez-González B, Luna R, Aguilera A (2011) Genome Instability and Transcription Elongation Impairment in Human Cells Depleted of THO/TREX. PLoS Genet 7: e1002386 doi:10.1371/journal.pgen.1002386.g010. 22144908 10.1371/journal.pgen.1002386 PMC3228816

[41] Tsuzuki T, Fujii Y, Sakumi K, Tominaga Y, Nakao K, et al (1996) Targeted disruption of the Rad51 gene leads to lethality in embryonic mice. Proc Natl Acad Sci USA 93: 6236–6240. 8692798 10.1073/pnas.93.13.6236 PMC39005

