# Correction: The *Caenorhabditis elegans* THO Complex Is Required for the Mitotic Cell Cycle and Development

**DOI:** 10.1371/journal.pone.0160138

**Published:** 2016-07-21

**Authors:** Maikel Castellano-Pozo, Tatiana García-Muse, Andrés Aguilera

The gene names nfx-1 and nfx-2 appear incorrectly throughout the article. The correct allele names should be nxf-1 and nxf-2 respectively.
