# Supplementary figures and images for: The Caenorhabditis elegans THO Complex Is Required for the Mitotic Cell Cycle and Development
Source: PLoS One. 2012 Dec 20;7(12):e52447. doi: 10.1371/journal.pone.0052447 (PMC3527488; doi:10.1371/journal.pone.0052447)

**A**

DAPI

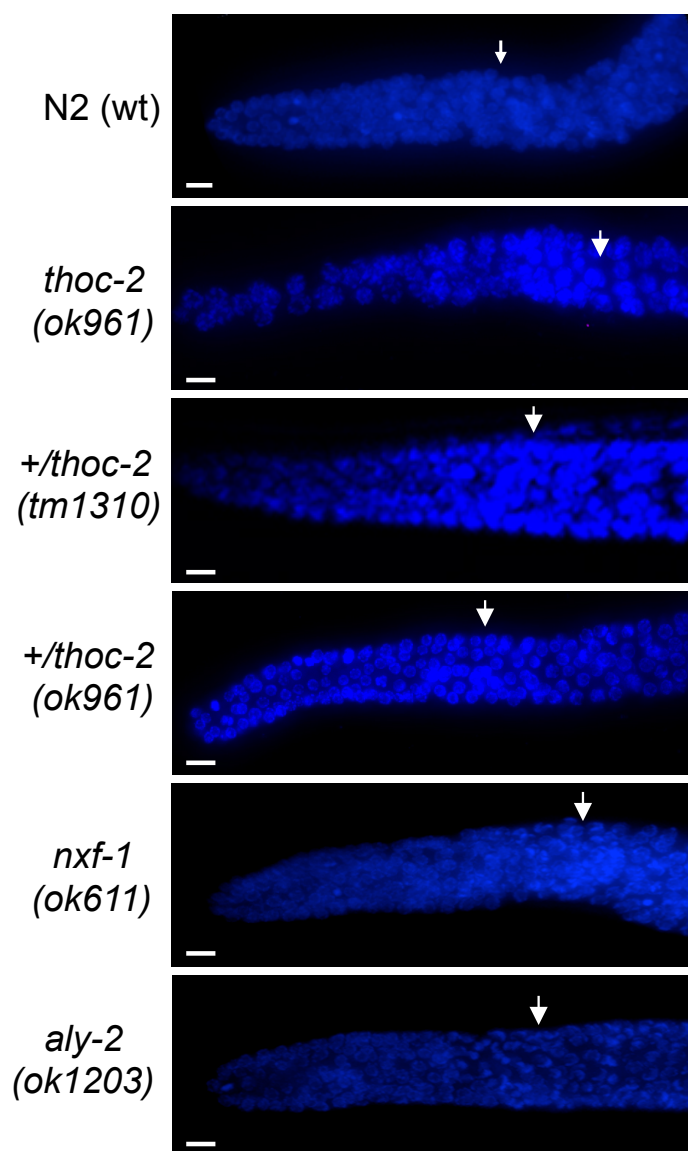**B**

mRNA

+DAPI

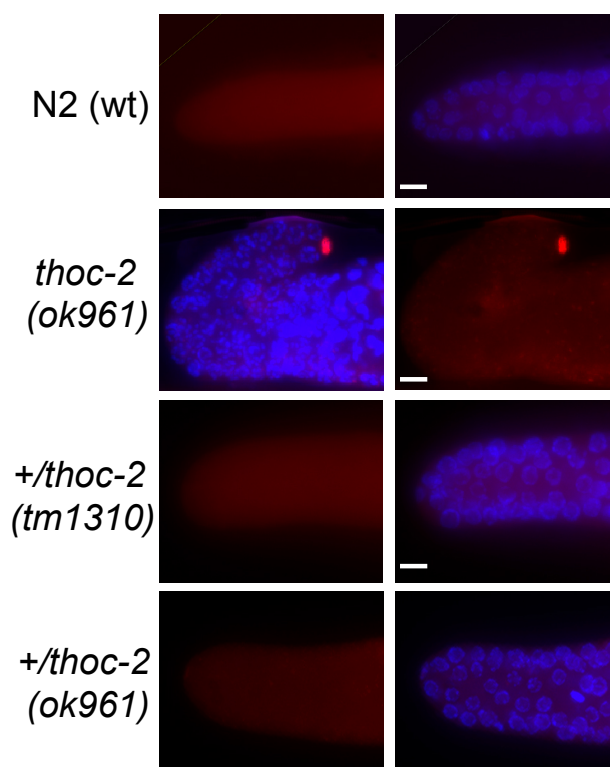**C**

mRNA

+DAPI

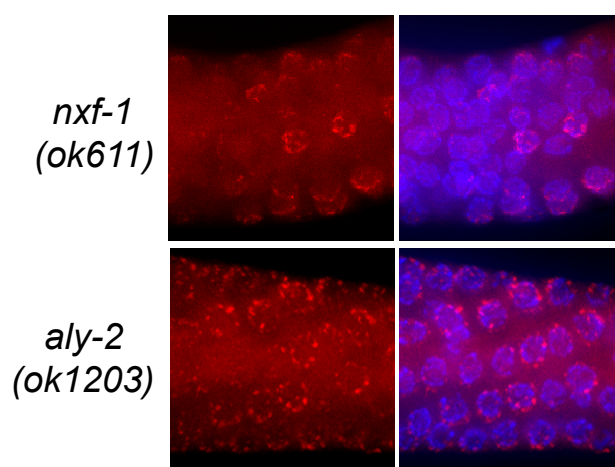

Supplement: Figure S1 — Normal mitosis in thoc-2 heterozygous worms. (A) Representative images of a single focal plane through the mitotic region of the germline from animals of the indicated genotype counterstained with DAPI. (B) Accumulation of mRNA visualized by FISH with a poly-dT oligonucleotide conjugated with Cy3 in germlines from animals of the indicated genotype image at the mitotic region. (C) Accumulation of mRNA visualized by FISH with a poly-dT oligonucleotide conjugated with Cy3 in germlines from animals of the indicated genotype image at the pachytene region. (PDF) [file pone.0052447.s001.pdf]

**A**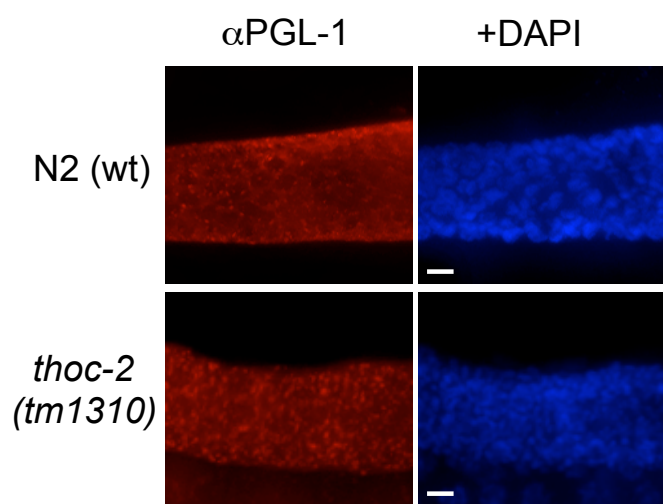**B**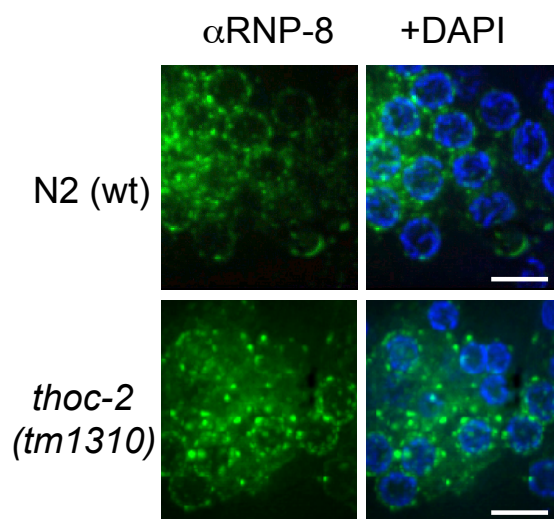**C**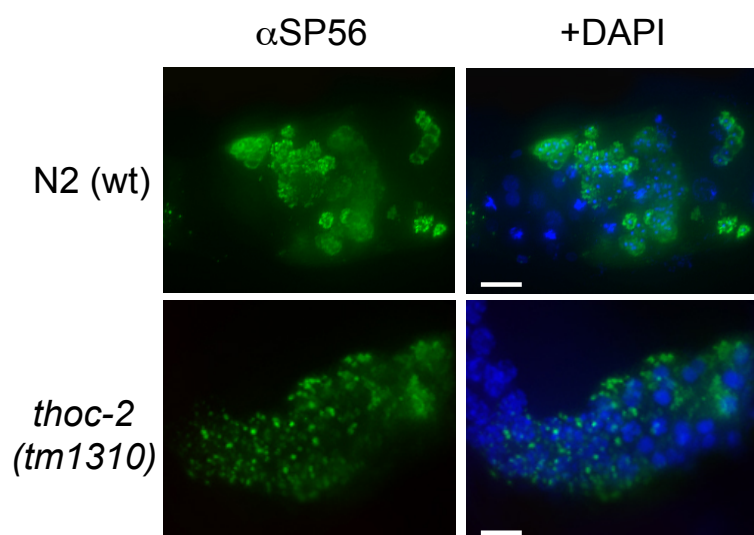**D**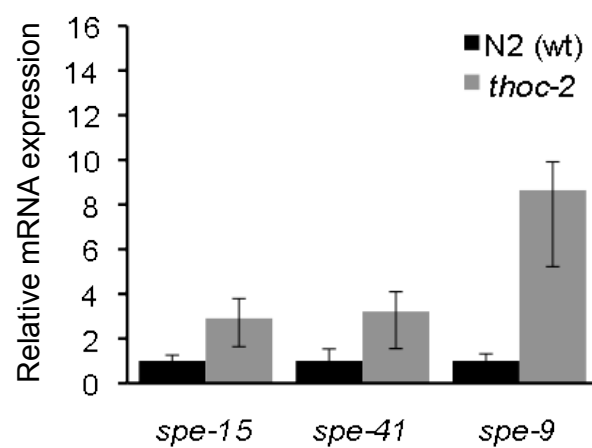

Supplement: Figure S2 — Normal expression and localization of meiosis markers in thoc-2 mutants. (A) Representative images of fixed pachytene nuclei from N2(wt) and thoc-2(tm1310) worms immunostained with the oogenesis marker PGL-1. (B) Representative images of fixed pachytene nuclei from N2(wt) and thoc-2(tm1310) worms immunostained with the oogenesis marker RNP-8. (C) Representative images of fixed pachytene nuclei from N2(wt) and thoc-2(tm1310) immunostained with the SP56 spermatogenesis marker. (D) RT-qPCR quantification of the indicated genes involved in subsequent steps of spermatogenesis in N2(wt) and thoc-2 mutant worms. (PDF) [file pone.0052447.s002.pdf]

DAPI (-HU)

DAPI (+HU)

N2(wt)

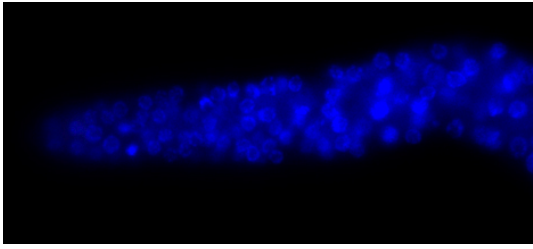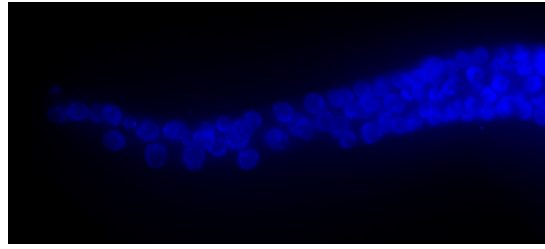

*thoc-2*  
(*tm1310*)

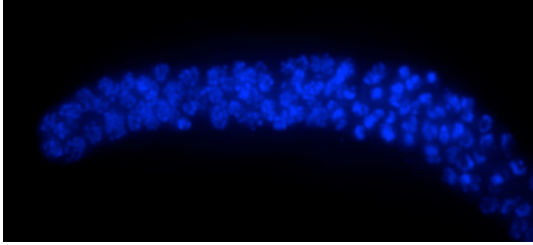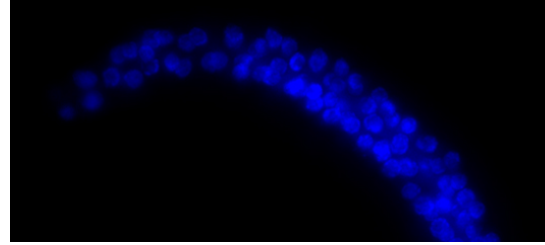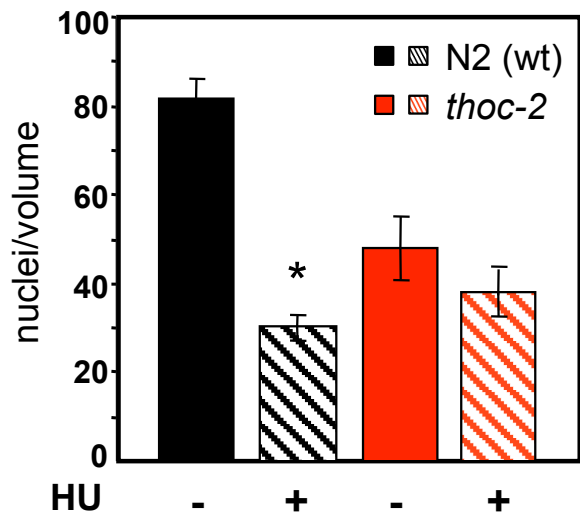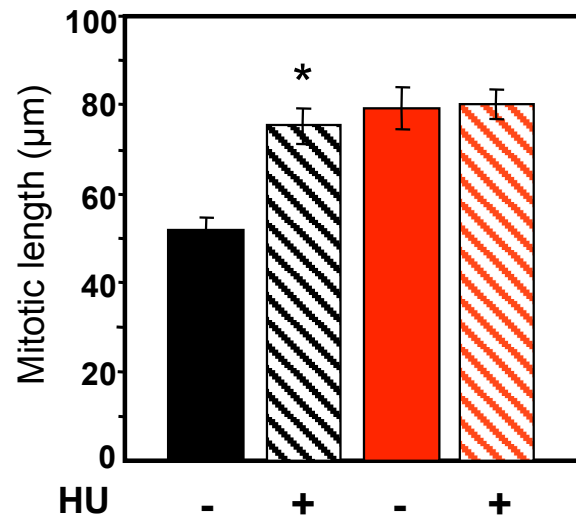

Supplement: Figure S3 — Response to replication stress in thoc-2 . Representative images of a single focal plane through the mitotic region of the germline from animals of the indicated genotype counterstained with DAPI and quantification of the number of mitotic nuclei and the mitotic region length. Error bars indicate standard errors of means (n = 30). Statistically significant differences versus the untreated N2(wt) (p<0.001) is indicated by an asterisk * (Student’s t-Test). (PDF) [file pone.0052447.s003.pdf]
