# Supplementary material for: The Caenorhabditis elegans THO Complex Is Required for the Mitotic Cell Cycle and Development
Source: PLoS One. 2012 Dec 20;7(12):e52447. doi: 10.1371/journal.pone.0052447 (PMC3527488; doi:10.1371/journal.pone.0052447)
Supplement: Table S1 — C. elegans THO complex comparison with mammalian THO complex members. (PDF) [file pone.0052447.s004.pdf]

Table S1. *C. elegans* THO complex comparison with mammalian THO complex members.

C. elegans	**Wormbase ID**	***H. sapiens***		
THOC-1	WBGene00020172	Thoc1	9.4e-78*	28**
THOC-2	WBGene00015813	Thoc2	9.4e-205*	76**
B0513.2	WBGene00007195	Thoc7	1.7e-16*	22**
Y32H12A.2	WBGene00021311	Thoc5	1.2e-33*	26**
THOC-3	WBGene00009341	Thoc3	7.4e-46*	32**

* BlastP e-value
** % Amino acid Identity
